# A new adhesive technique for internal fixation in midfacial surgery

**DOI:** 10.1186/1475-925X-7-16

**Published:** 2008-05-19

**Authors:** Kira Endres, Rudolf Marx, Joachim Tinschert, Dieter Christian Wirtz, Christian Stoll, Dieter Riediger, Ralf Smeets

**Affiliations:** 1 University Hospital Aachen, Dental Prosthetics Clinic, Medical Materials R&D Laboratory, Pauwelsstraße 30, 52057 Aachen, Germany; 2 CC&A Medical Components Ltd., Pauwelsstraße 19, 52057 Aachen, Germany; 3 University Clinic Bonn, Clinic for Orthopaedics and Accident Surgery, Sigmund-Freud-Straße 25, 53127 Bonn, Germany; 4 University Hospital Charité Berlin, Ruppiner Kliniken GmbH, Plastic Surgery Clinic for the Teeth, Mouth, Jaw and Face, Fehrbelliner Straße 38D, 16816 Neuruppin, Germany; 5 University Hospital Aachen, Departement for oral and maxillofacial surgery, Pauwelsstr. 30, 52057 Aachen, Germany

## Abstract

**Background:**

The current surgical therapy of midfacial fractures involves internal fixation in which bone fragments are fixed in their anatomical positions with osteosynthesis plates and corresponding screws until bone healing is complete. This often causes new fractures to fragile bones while drilling pilot holes or trying to insert screws. The adhesive fixation of osteosynthesis plates using PMMA bone cement could offer a viable alternative for fixing the plates without screws. In order to achieve the adhesive bonding of bone cement to cortical bone in the viscerocranium, an amphiphilic bone bonding agent was created, analogous to the dentin bonding agents currently on the market.

**Methods:**

The adhesive bonding strengths were measured using tension tests. For this, metal plates with 2.0 mm diameter screw holes were cemented with PMMA bone cement to cortical bovine bone samples from the femur diaphysis. The bone was conditioned with an amphiphilic bone bonding agent prior to cementing. The samples were stored for 1 to 42 days at 37 degrees C, either moist or completely submerged in an isotonic NaCl-solution, and then subjected to the tension tests.

**Results:**

Without the bone bonding agent, the bonding strength was close to zero (0.2 MPa). Primary stability with bone bonding agent is considered to be at ca. 8 MPa. Moist storage over 42 days resulted in decreased adhesion forces of ca. 6 MPa. Wet storage resulted in relatively constant bonding strengths of ca. 8 MPa.

**Conclusion:**

A new amphiphilic bone bonding agent was developed, which builds an optimizied interlayer between the hydrophilic bone surface and the hydrophobic PMMA bone cement and thus leads to adhesive bonding between them. Our *in vitro *investigations demonstrated the adhesive bonding of PMMA bone cement to cortical bone, which was also stable against hydrolysis. The newly developed adhesive fixing technique could be applied clinically when the fixation of osteosynthesis plates with screws is impossible. With the detected adhesion forces of ca. 6 to 8 MPa, it is assumed that the adhesive fixation system is able to secure bone fragments from the non-load bearing midfacial regions in their orthotopic positions until fracture consolidation is complete.

## Background

### Internal fixation of osteosynthesis plates in midfacial surgery

The objectives for the treatment of facial skeleton fractures are, besides restoring proper occlusion and the integrity of the nose and orbitae, the three dimensional reconstruction of the height, width, depth, and prominence of the midface [1,2]. These therapy objectives can only be realized by means of adequate immobilisation, using mini and/or microosteosynthesis plates and screws. This involves the repositioning of individual bone fragments to their accurate anatomical positions, and fixation with osteosynthesis plates. Therewith the use of rigid fixation devices has revolutionised the treatment of maxillofacial osteotomies and fractures [3].

### Standard systems with screws

**Figure 1:**
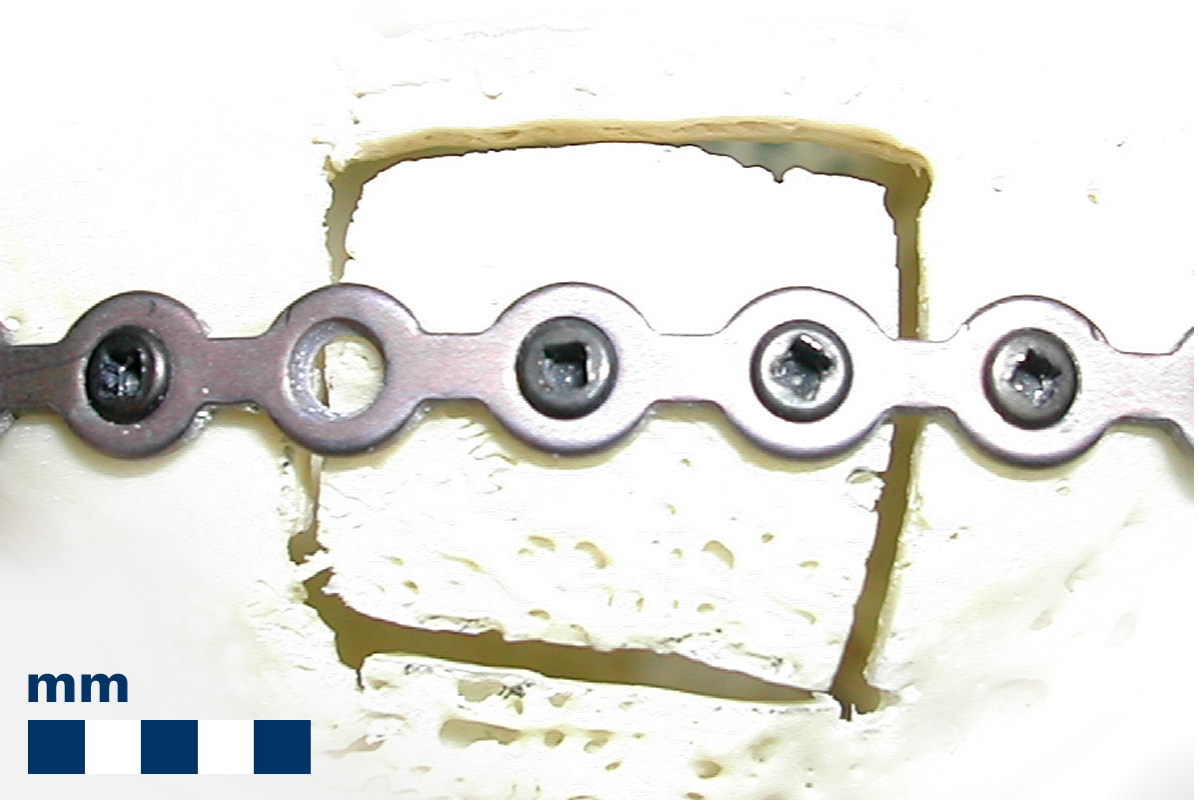
**Conventional fixation of an osteosynthesis plate with screws**. The conventional fixation of an osteosynthesis plate on a rabbit bone sample with a square cut damage of ca. 1 cm^2 ^as it is common in midfacial surgery. The plate is fixed to the bone by fastening screws in the osseous lamella to both the bone of sufficient cortical bone mass and to the fragile bone fragment.

The conventional fixation of osteosynthesis plates, made of titanium or an alloy of it, is carried out by either fastening screws into pilot holes in the osseous lamella, or inserting self-tapping screws. Figure 1 features the fixation technique with screws on a square-cut shaped rabbit bone fragment of ca. 1 cm^2^. This procedure requires areas of sufficient cortical bone mass. Otherwise, the presently proposed adhesive reposition method may be difficult to apply especially at sites where the boney structures are thin [3]. However, the non-load bearing areas of the nasoethmoidal, infraorbital, and frontal regions are extremely thin [2], such that only unfavourable options for fixation exist in those areas. Thus, the problems are still encountered in the clinical application of the plates [3]. The drilling of pilot holes, or fixation of screws in regions of thin cortical bone can cause further fractures, due to the force applied to the fragments [4-7], or, moreover, anatomical structures such as nerves, vessels or the roots of teeth can be injured when inserting screws [4,6]. In such cases, the conventional fixation of osteosynthesis plates by means of screw connection is not possible. The bone fragments cannot, then, be fixed in their orthotopic positions, and are left to heal indiscriminately. A prerequisite for the unhindered healing of bone following fracture, however, is the immobilization of the fragments sufficiently long for the osseous closure of the fracture gap [8-10]. Thus, the method of microplate screw fixation has different flaws, which affect the therapeutic effect and functional restoration of the patient [11]. This was the motive to develop an alternative fixation technique for mini- and microosteosynthesis plates in the craniofacial region [1].

### Alternative fixation by gluing

Glues have been around for a long time; 4,000 years ago in Egypt attempts were already made to take care of wounds by adhesive materials [11,12]. Objects joined by glues adhere as a result of two different physical forces: adhesion and cohesion [12]. The glue holds to the object by adhesion between the two dissimilar materials by intermolecular forces [12]. Nowadays adhesives are in use in all types of manufacture. Human structures can be repaired by adhesives including the fixation of osteochondral fragments, tendon and ligament ruptures and haemostasis as well as middle ear, facial laryngeal or tracheal surgery. All are challenges for the modern, active surgeon [11].

One promising alternative seems to be the use of adhesive systems for internal fixation that were already described in numerous in vitro and in vivo studies [1,3,13-16]. According to this studies it could be concluded that adhesive systems might be a useful alternative in bone bonding [3]. Thus, gluing is an attractive technique to fix bone fragments in orthopaedic and trauma surgery providing several advantages compared to nailing or screwing [1,16,17]. Therefore several preconditions have to be met by a bone adhesive for its all day clinical use. It must have appropriate adhesive properties, an adequate time of action and good short- and longterm biocompatibility without interference with the physiological fracture healing process [11,17].

Many efforts have been undertaken in the past to generate substances with adhesive properties for bone gluing purposes. Cyanoacrylates exhibited bad biocompatibility and high infection rates [17], whereas methacrylates and fibrin systems lacked sufficient adhesive stability [17,18]. Therefore, all these substances could not be established for all day clinical use [12,17-19]. Established adhesives, e.g. fibrin and protein-aldehyde systems, are indicated for soft tissue gluing but not for bone [17]. Fibrin sealant or fibrin glue (FG) as it is popularly known, has been used in a number of orthopaedic procedures to enhance osteogenesis in human maxillary and mandibular bone, in the fixation of osteochondral fractures, in spinal surgery and fixation of osteochondral fragments and bone chips [20]. Furthermore, this biological adhesive, a derivative of blood is widely used in surgery for their adhesive properties, hemostatic activity and wound healing process [20]. However their role in bone fracture healing or bone tissue response is not fully understood and controversies do exist, despite the fact that this biologic glue can be an interesting effective osteoinductive substitute [20]. Heiss et.al. described a newly developed alkylene bis(dilactoyl)-methacrylate as bone adhesive with some relationship to polymethylmethacrylate (PMMA) which has been used extensively in dentistry [18], and in orthopaedic surgery for anchoring of prostheses [17]. Preliminary in vitro data of this adhesive showed good biocompatibility in vivo without impairment of physiological fracture healing. It also shows good biodegradability characteristics [17] which are, however, not required in our study. By contrast, Grossterlinden et.al. observed an extensive tissue destruction after 6 months in all animals of a polymer group when alkylene bis(dilactoyl)-methacrylate was used for screw augmentation and for covering the osteotomy surface before osteosynthesis to analyze the influence of the material on bone healing [19]. This was attributed to a massive foreign body reaction at the histological level [19].

Nevertheless a longstanding history of research in this field a clinically applicable alternative was not found within the field of bone gluing. Former applications failed, because these adhesives were not tailored to the conditions met within the living organism [12]. However, the importance of this issue will persist into be more in the future and more studies about biocompatibility and bond strength of new bone adhesives will follow [12].

### Fixation with PMMA-Cement

**Figure 2:**
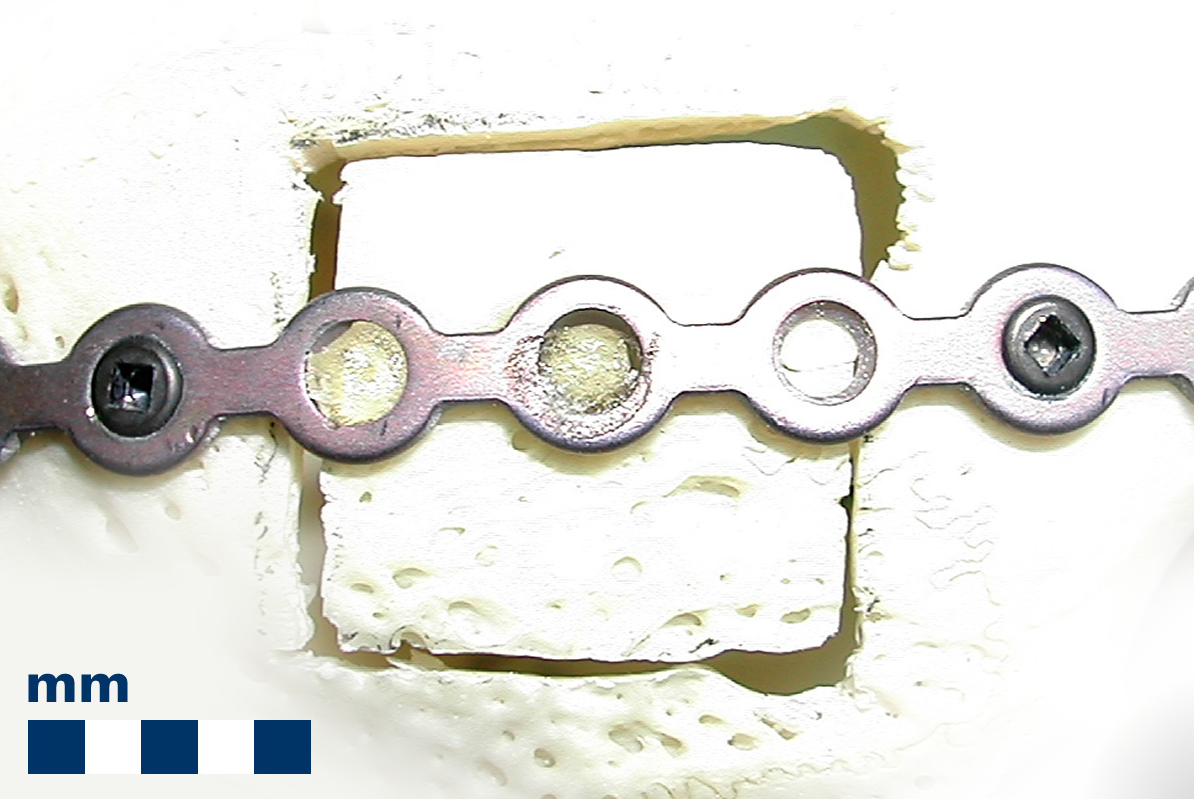
**Partially adhesive fixation of an osteosynthesis plate**. The new technique for the adhesive fixation of an osteosynthesis plate. The osteosynthesis plate is fixed on a rabbit bone sample with a square-cut damage of ca. 1 cm^2^. On sufficient cortical bone mass structures the plate is fixed with screws as usual. On the fragile bone fragment the plate is adhesively fixed to the bone of the sqare-cut damage with a PMMA bone cement and with an amphiphilic bone bonding agent as an intermediate layer.

In this article, we present an alternative technique using bone cement to affix thin cortical bone fragments to osteosynthesis plates in the surgical therapy of midfacial fractures. This procedure involves the conventional screw connection of the plate to thick cortical bone structures, while interjacent or delicate bone fragments are adhesively fastened to the plate with bone cement applied through the screw holes in the plate. Figure 2 represents this adhesive fixation technique also with a square-cut shaped bone fragment of ca. 1 cm^2^. The bone cement based on PMMA currently used with orthopaedic implants, for example for a hip or knee implant, is suitable for this application.

When implementing the adhesive fixation of osteosynthesis plates in midfacial surgery with a PMMA bone cement, one must be aware that the bonding mechanism that takes place differs from that when cementing a hip or knee endoprosthesis during orthopaedic surgery [1].

### PMMA-Cement in orthopaedics

Polymethylmethacrylate (PMMA) has been widely used in dentistry since 1930s, and in orthopaedic surgery, for the sealing of prostheses. It is not a true adhesive but interlocks well with cancellous bone. Charnley and Kettlewell pioneered the use of PMMA as a grouting agent in total hip replacement [21]. Up to now, there has been a constant increase in the use of that cementing system for implant anchorage, which shows the significance of this cementing technique. According to the Swedish Total Hip Replacement Register, the vast majority (93%) of primary total hip replacements (THRs) were performed using PMMA cement in the year 2000 [22].

In order to generate sufficient fixation between the bone and PMMA bone cement when cementing, for example, a THR on a sclerotic acetabulum bone structure, which is dense and plane, the sclerotic bone is cleared away until the subjacent spongious structure is exposed. Spongious bone has a structured porous surface area. When cementing the ductile bone cement is pressed into this porous bone structure. Thereby, the ductile bone cement fills the cavities of the porous bone and, after polymerisation, adequate fixation between the bone and bone cement is achieved, due to the retention forces of the rough surface [23,24].

### PMMA-Cement for adhesive fixation on non-retentive surfaces

The surface structure and associated wetting properties of the compact bones of the midface pose a problem for the fixation of reconstruction plates using the common PMMA bone cement. On the one hand, the compact bone of the midface has a cortical structure and is extremely dense and plane, lacking the surface cavities of spongious bone. Thus, the bone structure does not allow for the creation of the micro and macro retention forces between cement and bone described above for spongious bone, and the cement cannot anchor to the bone. On the other hand, the wetting properties of bone and PMMA bone cement are different [23,25]. Bone has hydrophilic properties and for water its wetting angle is, thus, lower than 90°, indicating that bone can be wetted by water very well. The monomers of PMMA bone cement, by contrast, have a hydrophobic character and a surface energy lower than that of bone [25]. Therefore, bone is not wetted by the monomers of ductile PMMA bone cement. As a result of the different wetting properties it is impossible to build adhesion forces between bone and bone cement [1,16,23,25,26]. Thus, if adhesion between bone and cement is to be created, the wetting properties of the two bonding partners must be adapted to each other.

### Bone bonding agent

The use of a bone bonding agent that is similar in composition to the dentin bonding agents having been in clinical use for years may offer a solution to the problem of incompatible wetting properties of the bonding partners [16,23-25,27]. The idea of such a bonding agent was patent-registered by Marx et al. in the year 2005. The invention concerns a novel coupling agent which hardens fast as it is photochemically polymerized for an efficient adherence between the bone cement and the bone surface during endoprosthetic implant grafting [27].

The dentin bonding agents are amphiphilic in nature and therefore able to bond with both hydrophilic dentin and hydrophobic composits, and according to several studies the use of dentin adhesives seemed to produce higher bond strength to bone than that attained with the cyanoacrylate adhesive [3,13-15]. Furthermore, cyanoacrylates exhibited bad biocompatibility and high infection rates as mentioned above [17].

Like dentin, bone also has a hydrophilic character, as a consequence of its high content of organic substances, especially collagen. In addition bone and dentin also have analogous chemical compositions [28] whereas the anorganic matrix of bone is ca. 67–70% by weight and that of dentin ca. 70% by weight, both consisting predominantly of Hydroxyapatit (Ca^2+^) [29-31]. The organic matrix of bone is ca. 22–23% by weight, while that of dentin is ca. 20% by weight, both composed mainly of collagen type I (NH_2_); the remaining weight percentage in both dentin and bone consists of water [29-31]. Furthermore bone cement, as a resin, is comperable to the composits, also hydrophobic in nature [23,25]. Thus, the field of dentistry has faced the same problem associated with a difference in wetting properties of the various components. This problem has been resolved with the use of dentin bonding agents. Consequently, the adhesion between bone and bone cement may nevertheless be achieved with the help of an interlayer system that forms a bridge between the bonding partners, accommodating the wetting properties of both partners [23,27].

**Figure 3:**
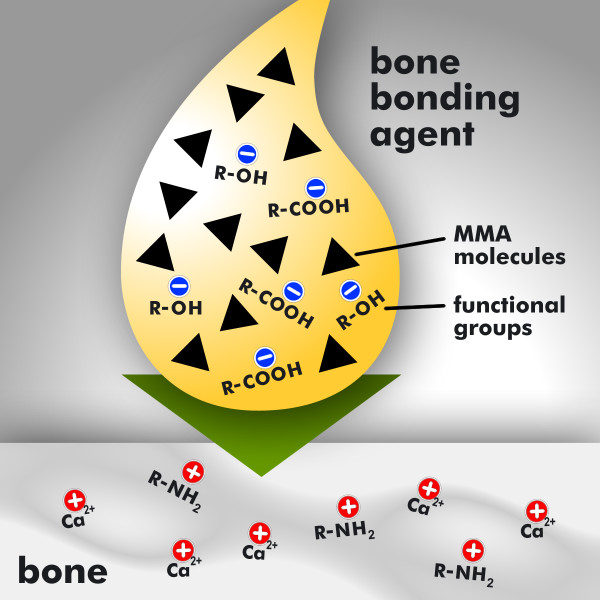
**Application of the bone bonding agent to bone**. Application of the amphiphilic bone bonding agent to the hydrophilic bone. The bone bonding agent (yellow drop) contains hydrophobic monomers like MMA molecules (marked with the black triangles) and functional groups like hydroxy groups R-OH and carboxy groups R-COOH, where R is a placeholder for the organic moiety. The bone (grey marked area at the bottom of the diagramm) contains calcium ions Ca ^2+ ^within its anorganic matrix and amino groups R-NH_2 _within its organic matrix; R is a placeholder for the organic moiety.

**Figure 4:**
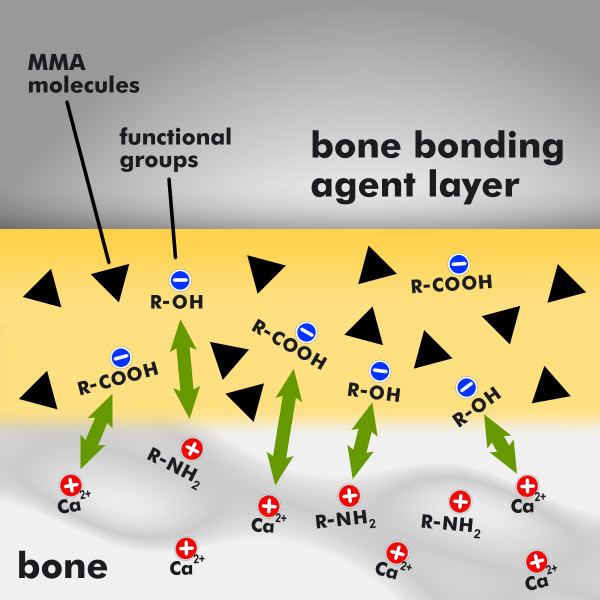
**Chemical bonding of the bone bonding agent to the bone**. With the infiltration of the applied amphiphilic bone bonding agent (yellow marked area) into the bone surface (grey marked area at the bottom of the diagram) a hybrid layer will be built. Therefore the hydrophilic monomers in the bone bonding agent serve to optimize the wetting of bone, which likewise has a hydrophilic character. Than, the functional groups of the bone bonding agent will build chemical bondings of electrostatic nature to bone. Thereby the hydrophilic carboxy groups (R-COOH) of the bone bonding agent are able to build a chemical connection to the calciumions (Ca^2+^) of the anorganic matrix of bone, while the hydrophilic hydroxy groups (R-OH) build a water-insoluble bond with the aminogroups (NH_2_) of the organic matrix of bone.

**Figure 5:**
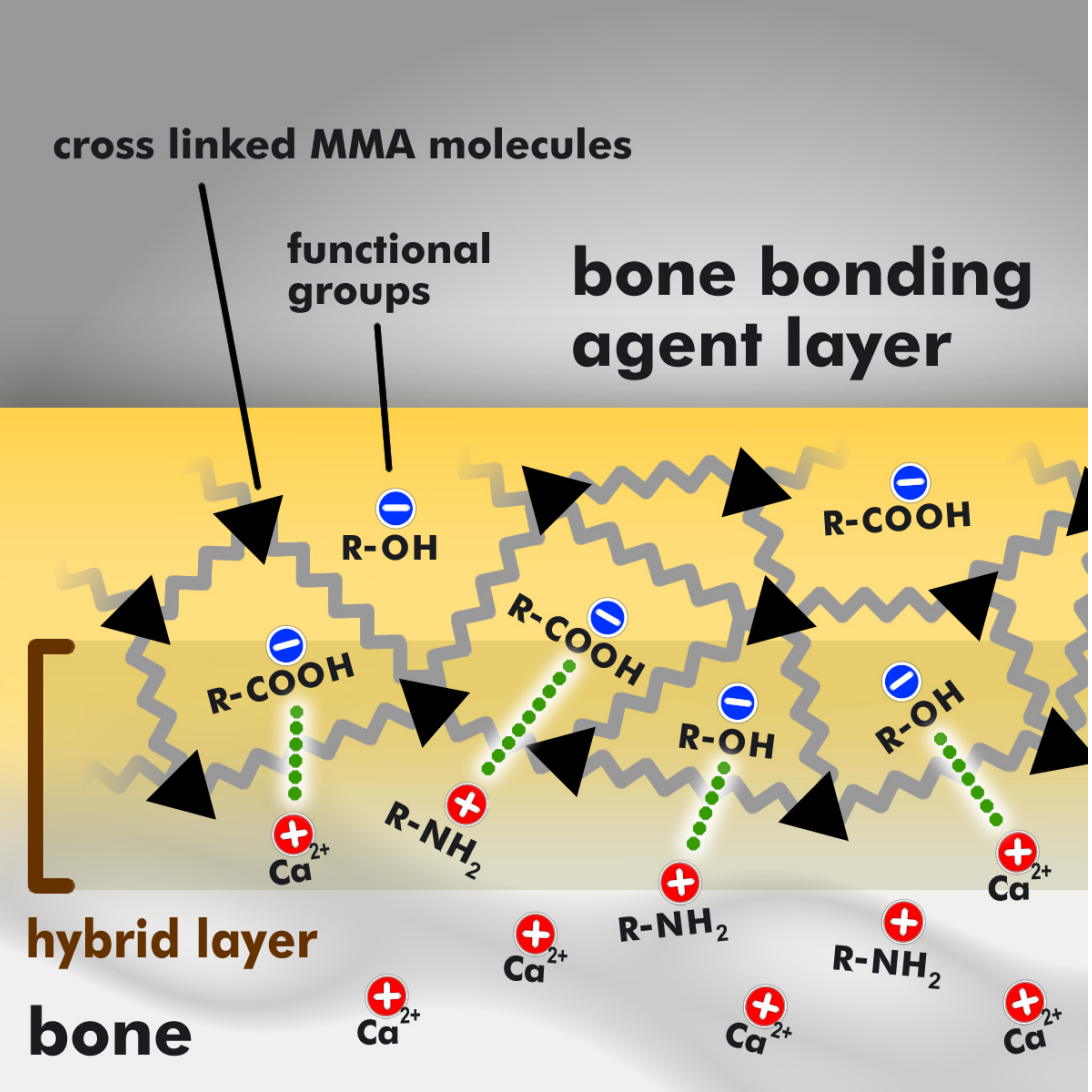
**Polymerisation of the bone bonding agent**. In addition to the chemical connection between the bone bonding agent and bone, the MMA molecules are polymerised with the help of UV-light building up crosslinks forming a coating to which bone cement can adhere.

Thus, analogous to the dentin bonding agents, the newly developed bone bonding agent is amphiphilic in nature. This amphiphilic bone bonding agent consists mainly of monomers that possess both hydrophilic and hydrophobic properties. The bone bonding agent as it is shown in Figure 3 (application of the bone bonding agent, yellow drop) contains hydrophobic monomers like MMA molecules and hydrophilic functional groups like hydroxy groups R-OH and carboxy groups R-COOH, where R is a placeholder for the organic rest. After the application of the bone bonding agent, it will infiltrate the bone surface, building up a hybrid layer as it is shown in Figure 4. Therefore the hydrophilic monomers in the bone bonding agent serve to optimize the wetting of bone having a hydrophilic character. Than, the functional groups of the bone bonding agent will build chemical bondings of electrostatic nature to bone. Thereby the hydrophilic carboxy groups (R-COOH) of the bone bonding agent are able to build a chemical connection to the calcium ions (Ca^2+^) of the anorganic matrix of bone, while the hydrophilic hydroxy groups (R-OH) build a water-insoluble bond with the aminogroups (NH_2_) of the organic matrix of bone. In addition the bone bonding agent contains photoinitiators for curing with UV light. After curing, the applied amphiphilic interlayer forms a coating, as shown in Figure 5, to which bone cement can adhere. In order to optimize the wetting of bone cement, having a hydrophobic character, the bone bonding agent contains hydrophobic monomers [16,27].

### Modified PMMA-Cement

The standard PMMA bone cements used in orthopaedics are solely self-curing, meaning that polymerisation takes place as a result of a chemical reaction when powder and liquid components are combined. This self-curing polymerisation process, as used in orthopaedics, takes a total of 10 to 15 minutes, whereby the initial phase, during preparation and implantation of the prosthesis, proceeds slowly and the final phase, following implantation, is more rapid. For cementing osteosynthesis plates in midfacial surgery, however, it is more desirable to have control over the initiation and duration of polymerisation. In contrast to the implantation of orthopaedic prostheses, the length of surgical fixation procedures using osteosynthesis plates is variable according to the number of plates required, and the length of time necessary to apply the cement through the screw holes of the plates.

In order to lengthen the processing time, standard PMMA bone cement was modified with an inhibitor to delay the chemical reaction time and, thus, the polymerisation. In order to allow for the initiation of polymerisation to be determined individually through the use of UV light, as it is desired in the fixation of osteosynthesis plates, standard PMMA bone cement was modified with a photoinitiator.

## Methods

In-vitro experiments were carried out using bovine bone samples from the femoral diaphysis. The bones were sawed into slices and the bone marrow was removed. During preparation, the fresh bone was kept moist with 0.9 weight-% NaCl-solution and was deep frozen for storage. Metal plates of titanium alloy TiAl6V4 were designed with a size of 30 × 5 × 1 mm, similar to the osteosynthesis plates used in surgery. The screw holes had a diameter of 2 mm, and were spaced at a distance of 10 mm. The bone bonding agent was prepared, as above-mentioned, using amphiphilic monomers and photoinitiators.

Before cementing the metal plates to the bone, the periost was removed using a raspatory. The bone bonding agent was then applied with sterile cotton wool and spread onto the bone surface, and the polymerisation of the bone bonding agent was induced with UV light. The metal plates were then affixed to the bone samples using bone cement, punctiformally applied through the screw holes. Subsequently, the bone cement was cured using UV light and the samples were stored for 1 to 42 days at 37 degrees C in two groups of 20 samples, with group A under moist conditions at 100% humidity, and group B completely submerged in a 0.9 weight-% NaCl-solution. Tension tests according to DIN EN ISO 527-1 were carried out at day 1 and day 42. Further samples, prepared according to the described procedure, were tested directly following cementation in order to determine the approximate primary stability of the plates. A reference group consisting of samples prepared without the bone bonding agent was also tested directly after cementation.

Strain-extension-diagrams of the results were recorded, whereby the detected stress σ, a result of the force F relating to the adhesive cross-section, was graphed in dependence of the strain ε on the sample. The maximum stress, σ_max_, corresponds to the ultimate strength σ_fracture _and, thus, to the adhesive strength of the sample.

## Results

### Adhesive strengths of cemented plates

**Figure 6:**
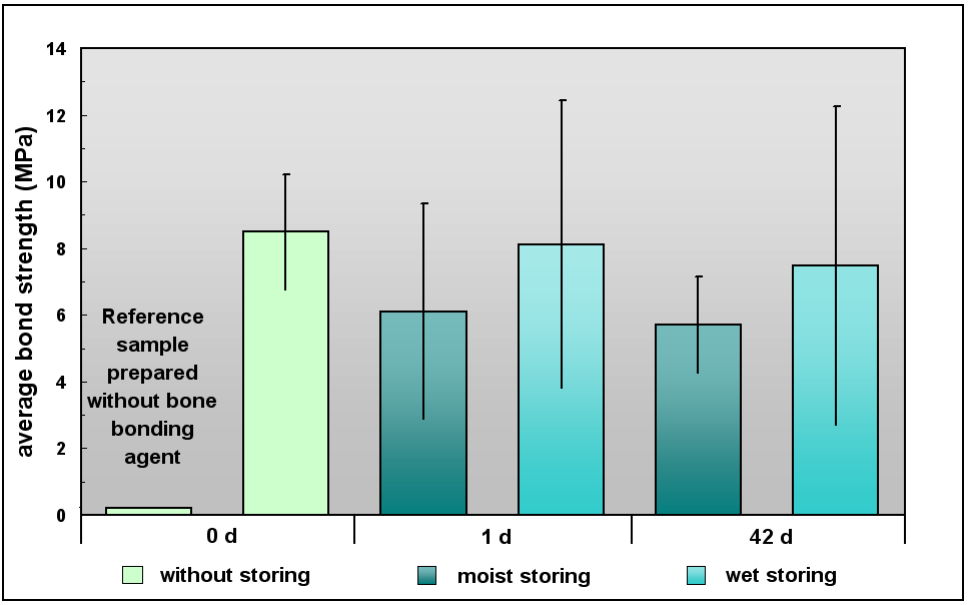
**Adhesive bonding strength of cemented osteosynthesis plates**. Average adhesive bonding strength and standard deviation measured in tension tests. The primary stability of the adhesive fixed plates is determined directly after cementation (without storing the samples, 0d). Samples prepared with the amphiphilic bone bonding agent (light green column on the right side) and, as a reference, without the bone bonding agent (light green column on the left side). The diagram also shows the effects on the adhesion strength of both moist storage (dark blue columns) and wet storage (light blue columns) for 1 and 42 days at 37 degrees C.

Figure 6 features the average adhesive strengths and standard deviations computed for the samples prepared with the amphiphilic bone bonding agent (light green column on the right side) and without it (light green column on the left side). Figure 6 also shows the effects on the adhesion strength of both moist storage (dark blue columns) and wet storage (light blue columns) at 37 degrees C whereas the samples were stored for 1 or 42 days. Adhesion forces which were detected directly after cementation in order to determine a value for the primary stability of the adhesively fixed plates were significantly lower for the plates fixed without the bone bonding agent of ca. 0.2 MPa than those of the samples prepared with the bone bonding agent of 8.5 ± 1.7 MPa. The adhesion forces for the reference samples, cemented without conditioning the bone, were nearly undetectable, indicating that adhesion did not take place between the cement and the cortical bone structure. In contrast, the average adhesive strength of ca. 8 MPa detected here directly after cementation for the samples conditioned with the bone bonding agent indicates that the adhesively fixed plates with an adhesive area of 3.14 mm^2 ^could be loaded with up to 2.7 kg before breakage occurred. Moist storage over 24 hours resulted in a decreased adhesive strength of 6.1 ± 3.2 MPa, as compared to 8 MPa primary stability within the reference group. No significant difference in adhesive strength was measured for the samples stored for 42 days under moist conditions with 5.7 ± 1.4 MPa. The samples stored submerged in a 0.9 % weight-NaCl-solution, however, showed a constant average adhesion strength at both 1 and 42 days of storage of 8.1 ± 4.3 MPa and 7.5 ± 4.5 MPa, respectively.

## Discussion

The newly developed bone bonding agent is advantageous for the clinical application of rigid fixation, in that it provides an alternative fixation technique when the use of screws is not possible. With the use of adhesive fixation, new trauma to delicate bone structures can be avoided during attachment to an osteosynthesis plate. Most new traumas result from the force applied when drilling pilot holes or fastening screws [5-7]. Up to now, the fixation of small or delicate bone fragments has been largely impossible, often leading to the healing of bone fragments in an undesirable anatomical situation [8]. With the new bone bonding agent and light-curing bone cement, the precise fixation of small and fragile fragments should become possible. Adhesive fixation with a cementing area of a few square millimeters affords an adequate supply of the surrounding periost, and undisturbed bone healing. Further, this adhesive fixation technique does not lead to the demineralisation of bone, in contrast to the adhesive fixation of synthetic fillings in conservative dentistry where the dentin surface is demineralized by the etching method. A further benefit of this drill-free osteosynthesis fixation system is the elimination of the risk of screw breakage or overwinding.

For regions of bone with a low cortical bone mass, that are not exposed to significant muscle traction or masticatory forces, average bond strengths of 6 to 8 MPa show potential for application in the adhesive fixation system. These results serve as an indication for further investigation. The next step should be to validate the results obtained here from *in vitro *bovine bone samples with *in vivo *animal experiments. For these *in vitro *tests, bovine bones were selected due to the fact that the ossification mode of the membranous bone of the bovine femoral diaphysis is comparable to the desmal ossification of the human viscerocranium [9]. Subsequently, the applicability of this newly developed system for use with human cortical bone should be examined.

Furthermore, the debonding of the plates shall be tested. In our in-vitro investigations a destructive method was used to determine the adhesive tensile strength. So, further examinations have to be done in order to develop a suitable method for the debonding of the plates. For example, one possibility could be to ream the PMMA cement from the screw holes of the plates.

Aside from the classic midfacial fractures, this technique could be used where other delicate bone fragments would previously have been tried to secure using screws, such as in cases of corrective osteotomy for craniosynostosis, for fractures of the orbital walls, or anterior walls of the maxillary and frontal sinuses, for periimplantation defects, or with the use of distractors.

### Bone bonding agent

Many efforts have been undertaken in the past to generate substances with adhesive properties for bone gluing purposes. Cyanoacrylates exhibited bad biocompatibility and high infection rates, whereas methacrylates and fibrin systems lacked sufficient adhesive stability [17,18]. A new class of bone adhesives based on alkylene bis(dilactoyl)-methacrylates may meet the requirements to bridge the gap between bench and bedside [17,19], however, the long-term biocompatibility as well as the combination with a copolymer that is used for both fragment adaption and implant fixation has to be investigated [19]. The long-term results obtained from the study of Grossterlinden et.al. suggest that (i) short-term observation not always allow valid conclusions regarding the biocompatibility of biomaterials, (ii) that biocompatibility might vary between species, and (iii) that the polymer based on alkylene bis(dilactoyl)-methacrylate used in this setting, although previously attributed to be a good candidate for clinical use in patients, does not meet the necessary criteria and tremendously interferes with the physiology of skeletal repair [19].

**Figure 7:**
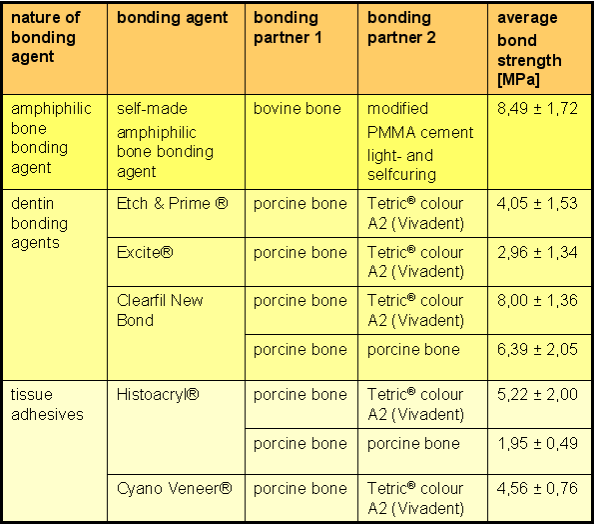
**Bonding strengths overwiev**. Average adhesive bonding strengths and standard deviations detected in a study of Maurer et al. using different kinds of bonding agents like dentin bonding agents (Excite, Clearfil New Bond, Etch & Prime^®^3.0) and tissue adhesives (Cyano Veneer^®^, Histoacryl^®^) [3, 29, 31], compared with the average bonding strength reached with the self-made amphiphilic bone bonding agent. The bonding partners variations: porcine bone/Tetric^® ^colour A2, porcine bone/porcine bone and bovine bone/PMMA cement. Tensile strength tests were done at day 0 (day of preparing the samples, without storing the samples).

Preliminary data confirmed the adhesive potential of different dentin bonding agents to bone and their efficacy in bone fixation under in vitro conditions. Maurer et al. compared tensile bond strength of three dentine adhesive systems (Excite, Clearfil New Bond, Etch & Prime^®^3.0) and two cyanoacrylate adhesives (Cyano Veneer^®^, Histoacryl^®^) to porcine bone in vitro [29,31]. The tensile bond strengths were measured 15 min after application and after light curing of the composite material Tetric ^® ^Ceram (colour A2), without storing the samples [29]. The measured tensile bond strengths are shown in figure 7; Clearfil New Bond showed significantly higher bond strength than the other four adhesives. The outcomes of the authors'study (day 0) are also added in figure 7 wheras the PMMA cement was equated with the composit. Further investigations of Maurer et al. and Bekes et al. compared the tensile bond strengths attained between bone and bone using two different adhesive systems (Clearfil™ New Bond and Histoacryl^®^) in vitro on porcine bone samples. The tensile bond strength was measured 15 min after application [3,31]. The outcomes are also added in figure 7.

An one-to-one comparison to other studies would not be feasible since the various study designs of former investigations are largely different. The use of dentin varies from animal to human, the use of the composits are largely different, e.g. Z 100 composite (3M ESPE), Tetric^® ^colour A2 (Vivadent), Brilliant Dentin A2 (Coltène), also the adhesives differ from each study and the appliance of them. In addition the adhesion tests are varying from tension tests to shearbonding tests.

However, the most dentin adhesive systems include the etching technique. Etching the dentin surface means that the collagen structure would be exposed by removing the mineral part of the dentin structure so that the bonding agent could penetrate into the collagen matrix. The adhesive system presented in this study works without the etching technique. This should be preferable in order to preserve the bone structure and moreover for easy handling in surgery.

The bone bonding agent [27], which was primarily developed for application in orthopaedics [25,32], has been tested in previous experiments, e.g. in which two cortical bone samples were connected using PMMA bone cement [23,25]. In this investigation, sandwich samples (cortical bone/bone bonding agent/PMMA bone cement/bone bonding agent/cortical bone) were tested in a three-point bending test without storage and with long-term storage of up to 120 days [23,25]. These experiments demonstrated that the compound stability between bone cement and cortical bone was 50- to 100-times higher with the use of the bonding system[25]. While the use of PMMA bone cement is the standard in orthopaedic procedures, such as total hip replacement (THP) or total knee arthroplasty (TKA), the high level of anchorage achieved is due to the cancellous structure of the spongious bone which is filled by the cement. However, when an implantation is necessary in an area of dense cortical bone, the bone cement is then unable to penetrate into the bone structure and achieve anchorage [23,25]. It is necessary in such cases, to establish adhesive bonding between bone and PMMA bone cement, a situation which is normally impossible due to the difference in wetting properties between the two partners. The newly developed bone bonding agent, however, is able to accommodate both wetting properties, thereby leading to adhesive bonding [25,32,33]. Previous investigations by Marx et al. let to the development of this bone bonding agent in order to upgrade the insufficient anchorage of bone cement to bone due to the fact that the two main reasons of prosthesis loosening are hydrolysis and the insufficient anchorage of the PMMA bone cement to bone, reflected by aseptic prosthesis loosening [25,27,32-34]. Furthermore bone substance could be preserved if it is possible to anchor the implants with the help of adhesion forces, because there would be no longer the need of clearing away the quasicortical bonestructure, which is, especially in the view of revision surgery very important to be conserved. Therefore in-vitro studies were designed with the cadavers of sheeps. Plasma activated acetabulum cups made of polyethylene were implanted into the acetabular cavity of those cadavers using the current cementing technique with and without the newly developed bone bonding agent [25,33]. The achieved bonding strengths were determined in torsional-turn out tests; the compound stability showed in mean a 1.8-fold increase of the interface strength in case of preconditioned acetabular cavities with the bonding system [25,33]. In further investigations animal testings were done on sheeps with conventional cemented hip arthroplasty stems with and without conditioning the bone with the new bone bonding agent [32,34]. In this investigation all stems of the verum group showed firm bonding of cement to bone, while in 7 of the 10 controls the stems with adherent cement could be easily pulled out off the bony implant bed. When preconditioned with the amphiphilic bonder, cemented stems showed a markedly higher adhesive strength to the cancellous bone without signs of inflammation or neoplasia. Thus, the bonder was biocompatible. The conclusion of this study: this procedure might offer enhanced longevity of cemented femoral revision stems in hip arthroplasty [34]. These in-vivo-experiments also demonstrated that the application of this newly bone bonding agent in vivo can achieve adhesive bonding between bone and bone cement. The use of the bone bonding agent had the effect of no debonding in the interface bone and PMMA bone cement in every case, whereas in seven of ten cases debonding comes out when the bone bonding agent was not applied [32,34].

The bones of the midface are cortical in structure and, therefore, dense and smooth. The need for an alternative fixation of osteosynthesis plates in this midfacial region motivated the application of the developed bone bonding agent for the adhesive fixation of the plates. Modification of this agent into a "one bottle" adhesive system would allow simple application of bone bonding agent in clinical use, and thus the fixation of osteosynthesis plates by means of bone cement. The present study demonstrated that the adhesive fixation of osteosynthesis plates by means of bone cement without conditioning the bone with bone bonding agent does not give rise to adhesion forces between the PMMA bone cement and cortical bone, having produced a nearly non-detectable adhesive strength of ca. 0.2 MPa. However, the *in vitro *tests involving the use of bone bonding agent showed an average adhesive strength of ca. 6 MPa with an adhesive area of 3.14 mm^2^, confirming the results of previous examinations. The adhesive bonding between bone and a PMMA bone cement is possible, and can doubtlessly be applied clinically. The adhesive strength of ca. 8 MPa (primary stability) allowed for the adhesively fixed plate to be loaded with ca. 2.7 kg before bond failure was noticable in the interface between bone cement and bone. The long-term stability of ca. 7 MPa was demonstrated after 6 weeks of storage in a 0.9 weight-% NaCl-solution at 37 degrees C. This translates into the ability of the adhesive bond to withhold up to 2.3 kg of weight. This is a favorable result, given the extremely small adhesive area.

The high level of standard deviation computed (20–64 %) is easily understandable, considering that bone is a biological material with variations in chemical and biomechanical properties among individuals and changing surface areas. This naturally applies to both human and animal bone. The bone samples used in this study were obtained from the same slaughterhouse, but were obtained on different days, and from different cattle, representing a source of error that cannot be controlled when testing biological materials. The detection of such high levels of standard deviation is not a new phenomenon, having been described as early as 1968 by Ansell and Scales [35]. These investigators determined the need for a synthetic standard material for their investigations on the holding force of osteosynthesis screws using rip-off tests. They succeeded in demonstrating the same rip-off forces with both bone and the synthetic material, though the levels of standard deviation varied greatly [35]. The use of phenolic resin as a standard material has been confirmed and reconfirmed by various authors in the years 1972 and 1980 [36,37]. In 1998, Heidemann conducted a comparative study of the holding force of osteosynthesis screws using materials such as porcine bone, beech tree wood, and PVC [38]. This study also demonstrated extremely high standard deviation levels in the experiments with porcine bones, while the lowest levels of standard deviation were calculated with PVC. For the study presented here, however, no standard material could be used, as the experiments were designed to test the effectiveness of the bone bonding material, in light of the clinical application in midfacial surgery. The wetting properties of bone were an essential factor in this experiment, and could not have been replicated in a synthetic material.

### Modified PMMA-Cement

For the clinical application of adhesively fixing osteosynthesis plates in midfacial surgery, a UV light-curing PMMA bone cement was developed by adding a photoinitiator to the PMMA powder component. This modified cement allows for a surgeon to individually determine the point in time at which polymerisation begins, in contrast to standard PMMA bone cements where the polymerisation takes up to 15 minutes, which would cost the surgeon and his team precious time when adhesively fixating an osteosynthesis plate during midfacial surgery. With the addition of a photoinitiator to the PMMA bone cement, the polymerisation time can be reduced from 10–15 minutes to as low as 90 seconds. This represents a much more manageable time frame for the fixation of an osteosynthesis plate. The wavelength of UV light used in this experiment was ca. 400–500 nm which was appropriate to the photoinitiator used in this system. Further experiments might modify the wavelengths used, and perhaps achieve even shorter light-curing times.

## Conclusion

Contrary to the biologically and technically well-founded expectations that the adhesive bonding of PMMA cement to cortical bone is impossible, this *in vitro *study showed that this goal can be achieved. The conditioning of hydrophilic cortical bone with an amphiphilic bone bonding agent results in high adhesive strength to hydrophobic PMMA bone cement. This newly developed bone bonding agent is similar to the amphiphilic dentin bonding agents, which were made available in recent years. The amphiphilic dentin bonding agents allow the adhesive connection of hydrophilic dentin to hydrophobic composits. The reference samples presented in our investigations confirmed again that the adhesive bonding between hydrophilic and hydrophobic bonding partners is impossible when an amphiphilic bonding agent is not used. Conditioning of the bone with the bone bonding agent leads to the successful adhesive bonding of PMMA cement to cortical bone. These results lead research into the punctiformally adhesive fixation of osteosynthesis plates to cortical bone in midfacial surgery.

The new approach for the reconstruction of midfacial fractures presented here through *in vitro *experiments offers new possibilities for the fixation of osteosynthesis plate systems. The decrease in trauma to the bone is a distinct advantage of this system over the present techniques. This is especially true for fixation in regions of extremely low cortical bone mass that offer only limited possibilities for conventional fixation with screws. It is assumed that the adhesive fixation system developed here would be able to secure bone fragments from the non-load bearing midfacial regions in their orthotopic positions until fracture consolidation is complete. The extent to which the results obtained here on bovine bone *in vitro *can be applied to an *in vivo *system will be determined in future animal experiments. Subsequently, the applicability of these results for human cortical bone should be examined in clinical studies.

## Abbreviations

ca: circa; cm: centimeter; cm^2^: square centimeter; mm: millimeter; mm^2^: square millimeter; nm: nanometer; kg: kilogram; NaCl: sodium chloride; MMA: methylmethacrylate; PMMA: polymethylmethacrylate; PVC: polyvinyl chloride; MPa: Megapascal; C: Celsius; UV: ultra violet.

## Competing interests

The contribution of KE to the work was sponsored by Synthes GmbH; forthcoming animal experiments will be sponsored by Synthes GmbH, Basel, Switzerland. RM, JT, DCW, CS, DR and RS declare that they have no competing interests.

## Authors' contributions

KE conceived of the study, participated in the design of the study, carried out experimental work, and drafted the manuscript. RM concieved of the study, participated in the design of the study and coordinated the work. DCW conceived of previous investigations leading to this study and carried out previous clinical and experimental studies. DR and JT coordinated the work. RS arranged the clinical basics and drafted the manuscript. All authors read and approved the final manuscript.
